# Hepatitis B Infection Is Associated with Asymptomatic Malaria in the Brazilian Amazon

**DOI:** 10.1371/journal.pone.0019841

**Published:** 2011-05-18

**Authors:** Bruno B. Andrade, Cristiane J. N. Santos, Luís M. Camargo, Sebastião M. Souza-Neto, Antonio Reis-Filho, Jorge Clarêncio, Vitor R. R. Mendonça, Nívea F. Luz, Erney P. Camargo, Aldina Barral, Antônio A. M. Silva, Manoel Barral-Netto

**Affiliations:** 1 Centro de Pesquisas Gonçalo Moniz, Fundação Oswaldo Cruz, Salvador, Brazil; 2 Faculdade de Medicina da Bahia, Universidade Federal da Bahia, Salvador, Brazil; 3 Departamento de Saúde Publica, Centro de Ciências da Saúde, Universidade Federal do Maranhão, São Luis, Brazil; 4 Departamento de Parasitologia, Instituto de Ciências Biomédicas, Universidade de São Paulo, São Paulo, Brazil; 5 Faculdade de Medicina, Faculdade São Lucas, Porto Velho, Brazil; 6 Instituto de Investigação em Imunologia, Instituto Nacional de Ciência e Tecnologia (INCT), São Paulo, Brazil; Université Pierre et Marie Curie, France

## Abstract

**Background:**

Areas that are endemic for malaria are also highly endemic for hepatitis B virus (HBV) infection. Nevertheless, it is unknown whether HBV infection modifies the clinical presentation of malaria. This study aimed to address this question.

**Methodology and Findings:**

An observational study of 636 individuals was performed in Rondônia, western Amazon, Brazil between 2006 and 2007. Active and passive case detections identified *Plasmodium* infection by field microscopy and nested Polymerase Chain Reaction (PCR). HBV infections were identified by serology and confirmed by real-time PCR. Epidemiological information and plasma cytokine profiles were studied. The data were analyzed using adjusted multinomial logistic regression. *Plasmodium*-infected individuals with active HBV infection were more likely to be asymptomatic (OR: 120.13, P<0.0001), present with lower levels of parasitemia and demonstrate a decreased inflammatory cytokine profile. Nevertheless, co-infected individuals presented higher HBV viremia. *Plasmodium* parasitemia inversely correlated with plasma HBV DNA levels (r = −0.6; P = 0.0003).

**Conclusion:**

HBV infection diminishes the intensity of malaria infection in individuals from this endemic area. This effect seems related to cytokine balance and control of inflammatory responses. These findings add important insights to the understanding of the factors affecting the clinical outcomes of malaria in endemic regions.

## Introduction

Malaria continues to be a major health threat worldwide. Most regions highly endemic for malaria are also endemic for other infectious diseases, which may affect the malaria infection [1]. In this context, hepatitis B virus (HBV) infections are common in many of the malaria endemic areas. HBV induces a robust pro-inflammatory Type 1 immune response (Th1), which is important for *Plasmodium* clearance, but is also implicated in disease severity [2]. Whilst intriguing, little is known of the effects of HBV on the clinical presentation of malaria. Intrahepatic HBV replication is inhibited by *P. yoelii* infection in mice [3], and there is enhanced interferon (IFN)- γ and IFN-α/β production in the liver. In humans, results from a small investigation suggest that acute falciparum malaria modulates HBV viremia in patients with chronic HBV infection [4]. Moreover, a study performed in a Vietnamese hospital showed that patients with cerebral malaria had a slightly greater risk of registering positive serology for the HBV surface antigen (HBSAg) [5]; however, this study did not show a significant association between the overall risk of death caused by severe falciparum malaria and positivity for HBSAg [5]. There is no clear evidence that the clinical status of underlying hepatitis B-related liver disease is affected during malaria infection. In addition, the impact of HBV infection on malaria symptoms has not been adequately addressed. Here, we report a study aimed at comparing co-infected individuals to individuals with single infections of HBV or *P. falciparum* and/or *P. vivax* to evaluate how HBV infection influences the malaria burden in a region from the Brazilian Amazon.

## Methods

### Ethics statement

Written informed consent was obtained from all participants or their legally responsible guardians, and all clinical investigations were conducted according to the principles expressed in the Declaration of Helsinki. The project was approved by the institutional review board of the Faculdade de Medicina, Faculdade São Lucas, Rondônia, Brazil, where the study was performed.

### Study locality

A field observational study was performed between May 2006 and September 2007 in Rondônia State (10°12'43'' S; 63°49'44'' W), Brazilian Amazon. In this region, most malaria cases occur between April and September, with a high risk of infection [6], [7]. Rondônia accounts for 19% of malaria cases in the Brazilian Amazon (112,165 symptomatic cases in 2005), with an estimated prevalence of 8% [8]. *P. vivax* infection represents up to 80% of the malaria cases in Brazil, and *P. falciparum* infection accounts for 16.3% [9]. Vivax malaria presents high morbidity in endemic communities. Although rare, fatal cases *P. vivax* infection have been reported in Brazil [10], [11]. In contrast, asymptomatic infections by *P. falciparum* and *P. vivax* have been detected in epidemiological surveys in some regions of the Brazilian Amazon, indicating that clinical immunity does exist in both autochthonous and migrant populations [12], [13]. The incidence of HBV infection was 20.4 per 100,000 inhabitants in 2004 with a mortality rate of 7.43 per million, which is more than three times higher than the national mean of 2.37 [8]. Previous studies in the Brazilian Amazon have primarily tried to estimate co-infection rates [14].

### Study design and sampling

Both active and passive malaria case detection and diagnosis of HBV infection were performed. These included home visits in areas of high transmission, and study of individuals seeking care at the diagnostic centers of Brazilian National Foundation of Health (FUNASA). Individuals of both sexes, ranging in age from five to seventy years, who had resided in the endemic area for more than six months, were invited to participate. Exclusion criteria were as follows: documented viral hepatitis (A, C, and D), chronic alcoholism, human immunodeficiency virus type 1 infection, yellow fever, leptospirosis, cancer and chronic degenerative diseases, sickle cell trait and the use of hepatotoxic or immunosuppressant drugs. Twelve individuals withdrew consent and were excluded from the study. The study participants were interviewed and examined by a physician, and 20 mL of venous blood and thick blood smears were collected. Plasma samples and total blood were stored in liquid nitrogen. Total blood samples were used for molecular diagnosis of malaria and plasma samples analyzed in our laboratory facilities in Salvador, Bahia, Brazil.

**Figure 1:**
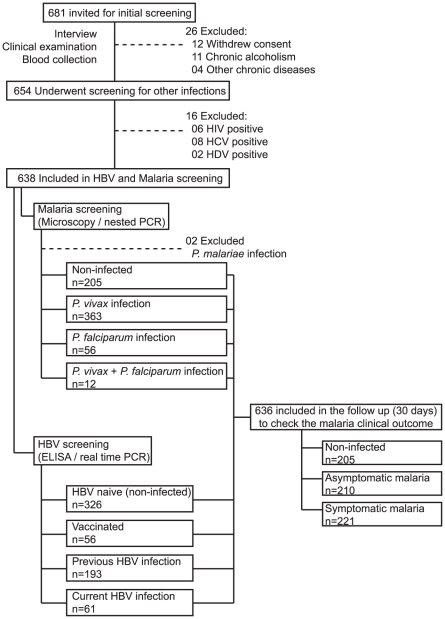
Screening and enrollment. The study participants were stratified according to the clinical presentation of *Plasmodium sp*. infection (non-infected, asymptomatic and symptomatic malaria). Simultaneously, the presence of HBV infection was verified to identify co-infections.

Malaria diagnosis was performed using microscopic examination of thick smears, and parasitemia (parasites/µL of blood) was calculated in positive cases. Nested PCR was performed as described previously [15], using whole blood samples from all individuals to confirm diagnoses. After obtaining the parasitological diagnosis, all positive cases were followed up for 30 days for the evaluation of malaria symptoms. Individuals who tested positive for *Plasmodium* infection without any presumptive malaria symptoms were considered asymptomatic, whereas cases with positive parasitological tests in the presence of symptoms were classified as symptomatic. Study individuals were then classified into three groups according to *Plasmodium* infection: non-infected (n = 205) or *Plasmodium*-infected (*P. vivax* and/or *P. falciparum*) and either symptomless (n = 221) or symptomatic (n = 210) (Table 1). Only two cases of *P. malariae* infection were detected, and these were excluded from the study (Figure 1).

**Table 1:** Baseline characteristics of the subjects.

	Current malaria			
Variables	Non-infectedn = 205	Symptomatic infectionn = 210	Asymptomatic infectionn = 221	P valueχ2
	n (%)	n (%)	n (%)	
**Gender**				0.227
Male	88 (42.93)	107 (50.95)	99 (44.80)	
Age (years)				<0.0001
5 to 15	14 (6.83)	25 (11.90)	1 (0.45)	
16 to 30	50 (24.39)	66 (31.43)	36 (16.29)	
31 to 59	98 (47.80)	83 (39.52)	145 (65.61)	
≥60	21 (10.24)	21 (10.0)	20 (9.05)	
**Years residing in the area**				<0.0001
≤2	44 (21.46)	56 (26.67)	34 (15.38)	
3 to 10	20 (9.76)	41 (19.52)	19 (8.60)	
>10	119 (58.05)	195 (92.86)	149 (67.42)	
**Residents per household**				<0.0001
1 to 5	141 (68.78)	113 (53.81)	147 (66.52)	
>5	42 (20.49)	82 (39.05)	55 (24.89)	
**HBV infection**				<0.0001
Non-infected	89 (43.41)	140 (68.30)	97 (43.89)	
Previous HBV	65 (31.70)	51 (24.30)	77 (34.84)	
Current HBV	29 (14.15)	04 (1.90)	28 (12.67)	
Vaccinated	22 (10.73)	15 (7.14)	19 (8.60)	
**Malaria diagnosis ‡**				<0.0001
Negative	205 (100)	-	-	
*P. vivax*	-	190 (90.48)	173 (78.28)	
*P. falciparum*	-	15 (7.14)	41 (18.55)	
*P. vivax* + *P. falciparum*	-	05 (2.38)	07 (3.17)	
**Malaria episodes**				<0.0001
None	24 (11.71)	25 (11.90)	03 (1.36)	
1 to 4	07 (3.41)	58 (27.62)	03 (1.36)	
5 to 10	29 (14.15)	49 (23.33)	07 (3.17)	
>10	123 (60.0)	63 (30.0)	189 (85.52)	
**Plasma IL-10 (pg/mL) §**				<0.0001
≤46	175 (85.37)	147 (70.0)	51 (23.08)	
>46	08 (3.90)	48 (22.86)	151 (68.33)	
**Plasma IFN-γ (pg/mL) §**				<0.0001
≤198	154 (75.12)	116 (55.24)	148 (66.97)	
>198	29 (14.15)	79 (37.62)	54 (24.43)	

A Chi-square test was performed to compare the distribution of each variable between the groups.

Individuals presenting AgHBS^−^/anti-HBS^+^/anti-HBc^+^ with no HBV DNA amplification by quantitative RT-PCR (qPCR) were considered to have previous HBV infection, those presenting AgHBS^+^/anti-HBS^−^ and detectable viremia by qPCR were considered currently infected with HBV and those with AgHBS^−^/anti-HBS^+^/anti-HBc^−^ were considered vaccinated against the virus.

‡Malaria diagnosis was based on light microscopy and confirmed by a nested RT-PCR molecular test, as described in methods.

§Cut-off IL-10 and IFN- γ plasma levels were determined by choosing the values that implied the highest likelihood ratio in discriminating asymptomatic from symptomatic malaria infection using a ROC analysis.

Diagnosis of HBV infection was performed at the State Central Laboratory (LACEN) of Salvador, Bahia, Brazil, using the AXSYM® automatic ELISA system (Abbott, Wiesbaden, Germany). All individuals were screened for HBSAg, total anti-HBS, total anti-HBc, anti-HBc IgM, HBeAg and anti-HBe IgG. We found that 326 individuals presented no markers of HBV exposure (HBSAg^−^/anti-HBS^−^/anti-HBc^−^), 193 presented markers of previous HBV infection (HBSAg^−^/anti-HBS^+^/anti-HBc^+^), 61 were currently infected (HBSAg^+^/anti-HBS^−^/anti-HBc^+^) and 56 were vaccinated (HBSAg^−^/anti-HBc^−^/anti-HBS^+^). All HBV infected individuals were positive for anti-HBc. No acute HBV infection was detected, as there were no individuals with anti-HBc IgM. Viremia was estimated by real-time PCR (COBAS® TaqMan® HBV assay) of all samples to confirm serological results. We also evaluated HBV-infected individuals for HBeAg and anti-HBe.

After serology for HBV infection, 636 individuals remained in the study. Plasma measurements of aspartate amino-transferase (AST), alanine amino-transaminase (ALT), total bilirubin, hemoglobin, fibrinogen and C-reactive protein (CRP) were made at the clinical laboratory of Faculdade São Lucas and at the Pharmacy School of the Federal University of Bahia, Brazil. A flow chart of the study is shown in the Figure 1. The baseline characteristics of the individuals are listed in the Table 1.

### Plasma cytokine measurement

IL-10, IFN-γ and TNF-α plasma levels were measured using the Cytometric Bead Array - CBA® (BD Biosciences Pharmingen, San Diego, CA, USA) according to the manufacturer's protocol, with all samples run in a single assay. The flow cytometric assay was performed and analyzed by a single operator, and standard curves were derived from cytokine standards.

### Statistical analysis

In the exploratory analysis of the data, frequency tables were constructed and the Chi-square test was applied to evaluate the association between qualitative variables. One polynomic (multinomial) logistic regression model was carried out because the response variable (current malaria infection) was classified into three groups (non-infected, asymptomatic infection and symptomatic). The following independent variables were included: HBV infection, malaria episodes, time residing in the area, residents per household, age, gender and plasma cytokine levels of IL-10 or IFN-γ. The threshold values of IL-10 or IFN-γ plasma levels, which discriminate asymptomatic from symptomatic malaria infection with a high likelihood ratio, were estimated using a ROC curve analysis to categorize the individuals according to cytokine levels and to perform the multinomial logistic regression (data not shown). Malaria parasitemia, cytokine plasma levels, and plasma levels of AST, ALT, total bilirubin, fibrinogen, and CRP were compared between groups using the Kruskal-Wallis test with Dunn's multiple comparisons. HBV DNA plasma levels were compared between groups using the Mann-Whitney test. The correlation between *Plasmodium* parasitemia and HBV viremia in co-infected individuals was checked using the Spearman test. We also plotted a non-linear curve fit to illustrate the general trend of this correlation. For each analysis, P<0.05 was considered statistically significant. The statistical analysis was performed using the software STATA 9.0 (StataCorp, TX, USA). The graphics were plotted using GraphPad Prism 5.0 (GraphPad Software Inc., USA).

## Results

### Baseline characteristics

A total of 636 individuals, out of 681 initially approached, were included. Individuals presenting no malaria infection differed from those presenting asymptomatic or symptomatic *Plasmodium* infection with regard to all variables studied, except for gender (Table 1). A total of 254 individuals presented serological markers of natural exposure to HBV, and 61 were experiencing infection at the time of the study (as identified by HBSAg and detectable viral load) (Table 1).

**Figure 2:**
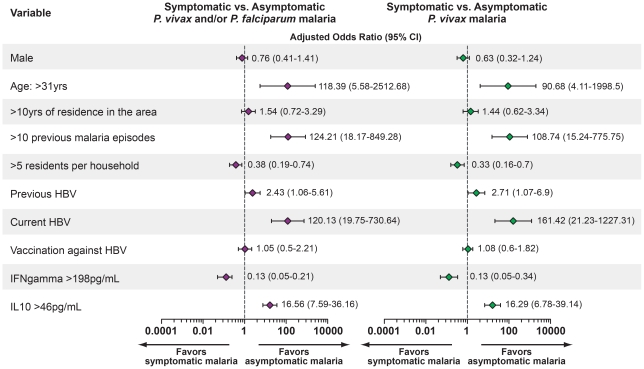
Adjusted multinomial logistic regression analysis of risk factors for asymptomatic malaria infection or for no malaria infection compared to symptomatic malaria. Symptomatic malaria infection was considered the primary outcome. Adjustment was performed for all variables presented. CI: Confidence interval. Individuals presenting AgHBS^−^/anti-HBS^+^/anti-HBc^+^ with no HBV DNA amplification by quantitative RT-PCR (qPCR) were considered to have previous HBV infection, those presenting AgHBS^+^/anti-HBS^−^ and detectable viremia by qPCR were considered currently infected with HBV and those with AgHBS^−^/anti-HBS^+^/anti-HBc^−^ were considered vaccinated against the virus. The statistical significance was estimated through multinomial logistic regression.

Individuals not included in the study (N = 45) were similar to those enrolled with regard to age, time of residence in the endemic area, number of residents per household, and number of previous malaria episodes, but were more likely to be female (P = 0.03) and test negative for *Plasmodium sp.* infection on thick smear examination (P = 0.001) and for markers of HBV exposure (P = 0.02). As expected, gender did not show any association with the absence of malaria or with asymptomatic infection (Figure 2).

### Impact of HBV exposure on malaria clinical presentation

To identify the variables independently associated with asymptomatic malaria, we performed a multinomial logistic model with adjustment for the all variables studied. We included variables previously described as protective factors against malaria [11], [15], [16], such as age, time of residence in the endemic area and number of previous malaria episodes. According to this analysis, age and increased number of previous malaria episodes were independently associated with asymptomatic infection (Figure 2). A high number of residents per household was associated with the occurrence of symptomatic malaria (odds ratio: 0.38, 95% CI: 0.19 to 0.74, P<0.0001; Figure 2). Vaccination against HBV did not influence the clinical presentation of malaria (OR: 1.05, 95% CI: 0.5 to 2.21; Figure 2). Previous HBV infection was associated with asymptomatic infection (OR: 2.43, 95% CI: 1.06-5.61, P<0.0001; Figure 2), and current HBV infection was even more robustly related to asymptomatic malaria (OR: 120.13, 95% CI: 19.75-730.64, P<0.0001; Figure 2).

We also tested whether the cytokine balance is associated with clinical immunity to malaria by examining the levels of two cytokines that indicate an inflammatory profile [11], [15], [16]. We found that increased plasma levels of IL-10 (values above 46 pg/mL) were independently associated with asymptomatic malaria (OR: 16.56, 95% CI: 7.59–36.16, P<0.0001), whereas higher levels of IFN-γ (values above 198 pg/mL) were related to the occurrence of symptomatic infection (OR: 0.13, 95% CI: 0.05–0.21, P<0.0001; Figure 2).

We then re-analyzed the data considering only the *P. vivax* cases, which represented 84.22% of the cases in our study (Table 2). The distribution of the epidemiologic, demographic and immunologic variables using only vivax malaria cases was similar to the previous analysis of both *P. vivax* and *P. falciparum* infections (Table 2). The exclusion of the *P. falciparum* cases did not alter the associations between HBV infections or the cytokine plasma levels with the occurrence of asymptomatic *Plasmodium* infection (Figure 2, left panel). Thus, the patterns of epidemiological associations did not change between the *P. vivax* and *P. falciparum* malaria cases. In addition, the systemic levels of both inflammatory patterns and cytokines did not significantly change between the infections from *P. vivax* and/or *P. falciparum* (data not shown), which allowed us to continue to analyze *P. vivax* and *P. falciparum* cases together.

**Table 2:** Baseline characteristics of the subjects, considering only *Plasmodium vivax* infections.

	Current vivax malaria			
Variables	Non-infectedn = 205	Symptomatic infectionn = 190	Asymptomatic infectionn = 173	P valueχ2
	n (%)	n (%)	n (%)	
**Gender**				0.0741
Male	88 (42.93)	102 (53.68)	77 (44.51)	
**Age (years)**				<0.0001
5 to 15	14 (6.83)	24 (12.63)	1 (0.58)	
16 to 30	50 (24.39)	63 (33.16)	25 (14.45)	
31 to 59	98 (47.80)	77 (40.53)	120 (69.36)	
≥60	21 (10.24)	18 (9.47)	17 (9.83)	
**Years residing in the area**				<0.0001
≤2	44 (21.46)	51 (26.84)	27 (15.61)	
3 to 10	20 (9.76)	38 (20.0)	17 (9.83)	
>10	119 (58.05)	93 (48.95)	119 (68.79)	
**Residents per household**				<0.0001
1 to 5	141 (68.78)	107 (56.32)	118 (68.21)	
>5	42 (20.49)	75 (39.47)	45 (26.01)	
**HBV infection**				<0.0001
Non-infected	89 (43.41)	132 (69.47)	74 (45.40)	
Previous HBV	65 (31.70)	47 (24.74)	64 (39.26)	
Current HBV	29 (14.15)	03 (1.58)	25 (15.34)	
Vaccinated	22 (10.73)	08 (4.21)	10 (5.78)	
**Malaria episodes**				<0.0001
None	24 (11.71)	25 (13.16)	03 (1.73)	
1 to 4	07 (3.41)	52 (27.37)	02 (1.16)	
5 to 10	29 (14.15)	45 (23.68)	04 (2.31)	
>10	123 (60.0)	60 (31.58)	154 (89.01)	
**Serum IL-10 (pg/mL) §**				<0.0001
≤46	175 (85.37)	139 (73.16)	42 (24.28)	
>46	08 (3.90)	43 (22.63)	121 (69.94)	
**Serum IFN-γ (pg/mL) §**				<0.0001
≤198	154 (75.12)	111 (58.42)	118 (68.21)	
>198	29 (14.15)	71 (37.37)	45 (26.01)	

A Chi-square test was performed to compare the distribution of each variable between the groups.

Individuals presenting AgHBS^−^/anti-HBS^+^/anti-HBc^+^ with no HBV DNA amplification by quantitative RT-PCR (qPCR) were considered to have previous HBV infection, those presenting AgHBS^+^/anti-HBS^−^ and detectable viremia by qPCR were considered currently infected with HBV and those with AgHBS^−^/anti-HBS^+^/anti-HBc^−^ were considered vaccinated against the virus.

§Cut-off IL-10 and IFN- γ plasma levels were determined by choosing the values that which implied the highest likelihood ratio in discriminating asymptomatic from symptomatic malaria infection using a ROC analysis.

### HBV exposure and laboratory assessment of malaria severity

**Figure 3:**
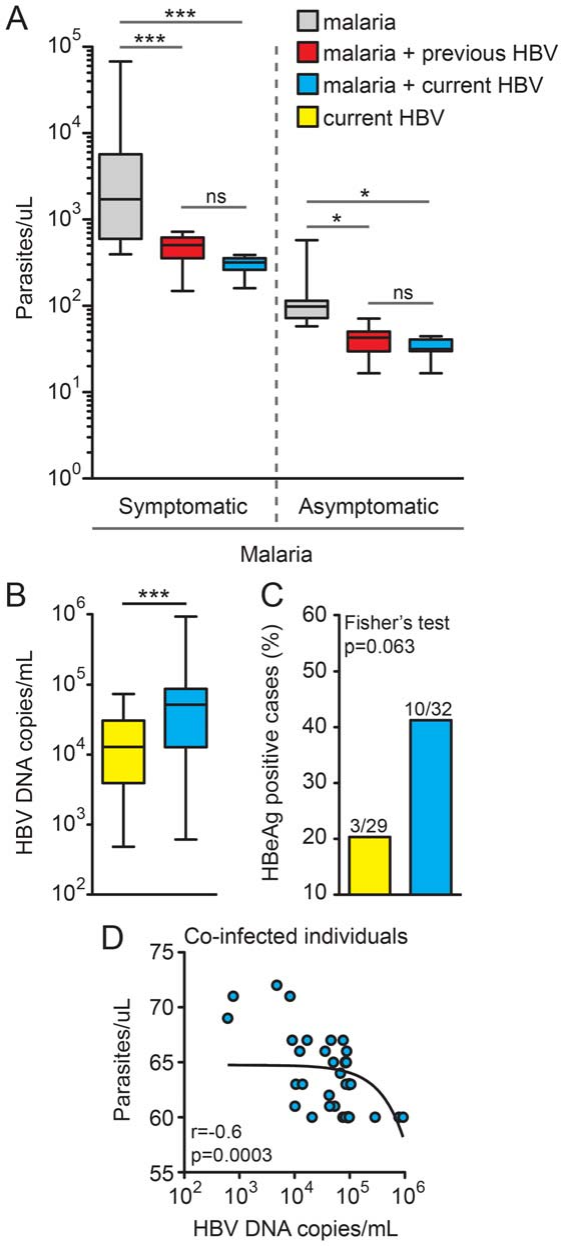
Individuals co-infected with *Plasmodium sp.* and HBV present lower parasitemia and higher viremia. (A) Individuals presenting with symptomatic or asymptomatic malaria were stratified according to the HBV status, and parasitemia levels were determined by light microscopy as described in the methods. The number of participants in each group is described in Table 1. Values were compared by the Kruskal-Wallis test with Dunn's multiple comparisons posttest. (B) HBV viremia was estimated by real-time PCR in both HBV infected (n = 29) or HBV-malaria co-infected (n = 32) individuals using the Mann-Whitney test and the percentage of HBeAg positive cases was also compared to these groups (C) using Fisher's exact test. Lines and boxes represent medians and interquartile ranges, and whiskers represent minimum and maximum values. (D) Spearman correlation between *Plasmodium* parasitemia and HBV viremia in co-infected individuals (n = 32). A non-linear curve fit was used to illustrate the general trend of the correlation. *P<0.05; ***P<0.0001.

To investigate the possible effects of active or previous HBV infection on the malaria severity, we compared malaria parasitemia between symptomatic or asymptomatic malaria cases, stratifying according to HBV status. Notably, in both symptomatic and asymptomatic individuals, active or previous HBV infection was linked to lower *Plasmodium* parasitemia (Figure 3A). Conversely, the number of HBV DNA copies/mL of blood in individuals infected with HBV alone was higher than in those co-infected with HBV and *Plasmodium* (P<0.0001; Figure 3B). In addition, these groups did not present significant differences in HBe antigen positivity (P = 0.063; Figure 3C), despite the observed trend favoring its association with HBV-malaria co-infection. Remarkably, in co-infected individuals, there was a significant negative correlation between *Plasmodium* parasitemia and HBV viremia (Spearman r = −0.6; P = 0.0003) (Figure 3D).

**Figure 4:**
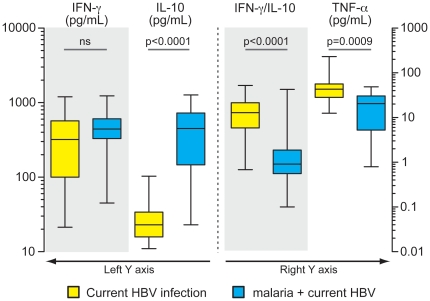
Co-infection with *Plasmodium* and HBV alters plasma cytokine profile. Plasma levels of IFN-γ, IL-10 and TNF-alpha were compared between individuals currently infected with HBV (n = 29) and those co-infected with HBV and malaria (n = 32). Lines and boxes represent median and interquartile range, and whiskers represent minimum and maximum values. Data were compared using a Mann-Whitney test. P values are shown in each graph.

**Figure 5:**
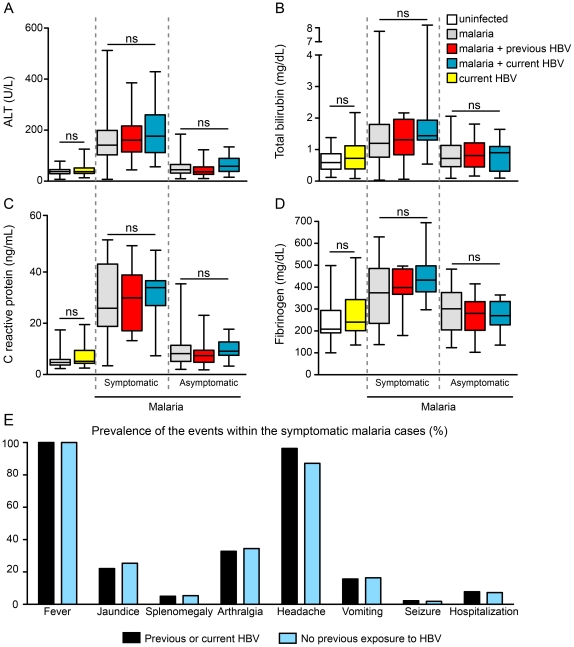
HBV infection did not alter either laboratory markers of organic dysfunction or the prevalence of symptoms in malaria cases. The study participants were stratified according to the clinical presentation of *Plasmodium* infection and also according to the HBV natural exposure and plasma levels of (A) alanine-aminotransferase (ALT), (B) total bilirubin, (C) C-reactive protein and (D) fibrinogen were compared using a Kruskal-Wallis test with Dunn's multiple comparisons. Lines and boxes represent medians and interquartile ranges, while whiskers represent minimum and maximum values. (E) The prevalence of diverse malaria-related symptoms was measured in individuals presenting with symptomatic malaria and compared between those with previous or current HBV infection and those with no markers of HBV exposure. The values were compared using Chi-square test and no significant differences were found.

The clinical presentation of both malaria and hepatitis B are correlated with cytokine balance. In the present study, individuals co-infected with HBV and *Plasmodium* presented similar systemic levels of IFN-γ compared with those infected solely with HBV (Figure 4). Nevertheless, co-infected individuals presented significantly higher plasma concentrations of IL-10 and slightly reduced levels of TNF-α compared with HBV mono-infected patients (Figure 4). Notably, HBV malaria co-infection was associated with reduced values of IFN-γ/IL-10 ratios (P<0.0001, compared with HBV mono-infection; Figure 4). Patients with symptomatic malaria presented with higher levels of AST, ALT, total bilirubin and CRP compared to both asymptomatic malaria and non-infected individuals (Figure 5A–D). In this scenario, previous or current HBV infection did not change the levels of these parameters, in addition to not modifying the overall prevalence of symptoms (Figure 5E).

## Discussion

This study is the first to provide strong evidence for the association between HBV and reduced malaria severity. *Plasmodium*-infected individuals with active or previous HBV infection were significantly more likely to be asymptomatic, to present with lower parasitemia and to have a decreased inflammatory cytokine profile. Co-infected individuals presented higher HBV viremia, and *Plasmodium* parasitemia was correlated to plasma HBV DNA titers. Additionally, the cytokine balance seems to be linked to disease severity, as individuals with asymptomatic malaria presented a reduced IFN- γ/IL-10 ratio. However, other factors in addition to cytokine profile must be involved in the reduced malaria severity in individuals with HBV, as the risk for asymptomatic infection was even higher when we analyzed the adjustment for plasma IL-10 and IFN-γ levels. It is possible that modifications in the hepatic microenvironment during HBV infection reduce the organ's susceptibility to *Plasmodium*.

This study confirms previous observations that asymptomatic *Plasmodium* infection correlates with increased age and longer periods of residency in regions where malaria is endemic [17], [18]. In addition, elevated IL-10 plasma levels correlated significantly with asymptomatic *Plasmodium* infection. Perhaps continued exposure to *Plasmodium* leads to IL-10 mediated immunomodulatory effects that limit immunopathology. IL-10 responses have been linked to human resistance to malaria [19]. HBV infection leads to increased IFN-γ levels [20], [21]. It has been shown that IFN-γ is important for *Plasmodium* clearance in the liver [22], in addition to its early importance for malaria clinical immunity [23]. In co-infected individuals, higher IFN-γ production could decrease parasitemia, leading to reduced malaria severity. However, *Plasmodium* infection is related to increased IL-10 plasma levels [24], [25]. Higher IL-10 production is related to reduced tissue damage in several diseases, including experimental [26] and human malaria [27], [28]. Polymorphisms associated with increased IL-10 production are related to increased severity of chronic HBV infection [29], [30]. In the present study, individuals presenting asymptomatic malaria displayed a lower IFN- γ/IL-10 ratio than their symptomatic counterparts. Thus, IL-10 may be linked to reduced malarial liver damage as well as increased viral load.

Other studies have addressed the association between HBV infection and *P. falciparum* but not *P. vivax* malaria. In one study, an association between HBV carriage and malaria severity was observed in children [31]. Another investigation suggested that chronic asymptomatic *P. falciparum* infection may be accompanied by sustained periods of HBV reactivation [4]. However, these data are limited in that only four patients were studied. More recently, one observational study in an Asian hospital proposed that chronic HBV infection exacerbates *P. falciparum* malaria [5]. However, that study analyzed only patients hospitalized with severe *P. falciparum* infection, ignoring the effect of HBV infection on uncomplicated malaria. In addition, the overall risk of death was not significantly higher in the co-infected patients [5]. Our study suggests that, in general, HBV does not worsen the pro-inflammatory cytokine parameters also altered by *Plasmodium* infection. In addition, exposure to HBV did not influence the frequency of hospitalization or even the prevalence of symptoms. The multivariate analysis revealed that other factors in addition to HBV infection might influence asymptomatic malaria. We propose that in older persons repeatedly exposed to *Plasmodium*, HBV exposure reduces parasitemia but does not alter organ dysfunction caused by malaria. Thus, a common mechanism affecting malaria immunity is postulated.

We recognize some limitations to this study. First, it is unclear whether the plasma cytokines reflect cytokine levels in the liver. Further studies are underway to address this issue. In addition, we did not screen our volunteers for helminth infections or glucose-6-phosphate dehydrogenase deficiency (G6PD). Helminth infection can affect malaria [32] by reducing the associated immunopathology [33], [34]. Recently, it was demonstrated that filarial infection modulates malaria-specific type-1 cytokine responses in an IL-10 dependent manner [35]. Our facilities were not prepared to perform fecal exams in all individuals. Local publications indicate that the prevalence of G6PD in Rondônia is about 3.3% [36], which would not interfere with our conclusions.

In conclusion, HBV infection seems to modify the physiology of the host's immune system, stimulating increased inflammatory responses, reducing *Plasmodium* parasitemia and possibly diminishing parasite burden. The results presented here need to be confirmed in future prospective studies.
